# Youth and climate crisis: participation and learning in complex, adaptive systems

**DOI:** 10.1007/s35834-024-00427-8

**Published:** 2024-07-09

**Authors:** Reingard Spannring, Natalia Waechter

**Affiliations:** 1 Institute for Educational Science, University of Innsbruck, Innrain 52a, 6020 Innsbruck, Austria; 2 Department of Educational Science, University of Graz, Merangasse 70, 8010 Graz, Austria

**Keywords:** Climate change, Crisis, Youth, Learning, Education, Complex adaptive system theory, Klimawandel, Krise, Jugend, Lernen, Bildung, Complex Adaptive System Theory

## Abstract

The Friday for Future school strikes have vividly demonstrated young people’s concern for the rapidly deteriorating life and survival chances of future generations of human and nonhuman beings. The young people’s ambivalence between despair and hope, feelings of powerlessness and empowerment challenge us to recognise the complexity and dynamic not only of ecosystems but of socio-ecological systems and to question the mechanistic and linear thinking often prevalent in youth research and education science. This thinkbite invites readers to consider young people’s experiences with the climate crisis and (climate change) education through the perspective of Complex Adaptive System Theory to open up learning space for more wholesome and creative responses to the challenge.

## Abstract (de)

Die Friday for Future Schulstreiks haben die Besorgnis junger Menschen über die sich rapide verschlechternden Lebens- und Überlebenschancen zukünftiger Generationen menschlicher und nicht-menschlicher Lebewesen anschaulich demonstriert. Die Ambivalenz der Jugendlichen zwischen Verzweiflung und Hoffnung, Gefühle der Machtlosigkeit und des Empowerments fordern uns heraus, die Komplexität und Dynamik nicht nur von Ökosystemen, sondern von sozio-ökologischen Systemen zu erkennen und das mechanistische und lineare Denken zu hinterfragen, das in der Jugendforschung und Erziehungswissenschaft oft vorherrscht. Dieser Denkanstoß lädt die Leser*innen dazu ein, die Erfahrungen junger Menschen mit der Klimakrise und der (Klimawandel‑)Bildung aus der Perspektive der Theorie komplexer adaptiver Systeme zu betrachten, um Lernräume für gesündere und kreativere Antworten auf die Herausforderung zu eröffnen.

## Introduction: the complexities of climate change

Global climate change increasingly affects marine, freshwater and terrestrial ecosystems and ecosystem services, water and food security, settlements and infrastructure, health and well-being, economies and culture, in particular through compound stresses and events (IPCC 2022). One important driver of anthropogenic change includes greenhouse gas emissions, which have doubled since 1980 raising average global temperatures by at least 0.7 degrees Celsius, increasing land and ocean surface temperatures, raising sea levels, changing the spatial and temporal patterns of precipitation, and increasing the frequency and intensity of El Niño events (IPCC 2002). The 2019 Emissions Gap Report (UNEP 2020) also clearly lays out the shortfall in carbon emission goals. Climate change (along with other forms of anthropogenic change such as deforestation, desertification, urban sprawl, biotic consumption and manipulation, Meyer 2006; quoted in Spannring 2021) is responsible for cascading effects through changes in the timing of reproduction in animals and plants, migration patterns of animals, length of growing seasons, species distributions and population sizes, frequency of disease outbreaks within and between species, and disturbances such as wildfires and the increase in pyro-convection and dry lightning, ecosystem composition and functionality (UNEP 2020). Ecosystems, species and wild populations are shrinking or vanishing (IPBES 2019), placing incredible pressure on the co-evolution of complex adaptive systems and associated micro-systems, that represent the overall Earth System.

Indeed, such pressures and perturbances call into question the ‘future of Earth’s environment and its ability to provide the services required to maintain viable human civilizations’ (Steffen et al. 2007, p. 614). Climate change impacts are concurrent and interact with other significant societal changes that have become more salient since IPCC’s fifth assessment report in 2014. They include “a growing and urbanising global population; significant inequality and demands for social justice; rapid technological change; continuing poverty, land and water degradation, biodiversity loss; food insecurity; and a global pandemic” (IPCC 2022, p. 45). These mutually reinforcing dynamics produce overarching future risks for low-lying coastal systems; terrestrial and ocean ecosystems; critical physical infrastructure, networks and services; living standards and equity; human health; food security; water security; and peace and migration. Risks are projected to become severe with increased warming and under ecological or societal conditions of high exposure and vulnerability (ibid., p. 55).

Climate change is the prime example par excellence for complex, socio-ecological systems. Complex Adaptive Systems Theory (CAS) has come to understand that mechanistic and linear relationships only apply to a minority of phenomena in a vastly nonlinear world (e.g., Prigogine and Stengers 1984; Waldrop 1992; Holland 1992; Kauffmann 1993). Today, many scientists work on *negative feedback dynamics* which keep a system within *safe operating parameters* and *positive feedback loops* in which a small increase in a variable can lead to a catastrophic runaway dynamic within the system, even to *tipping points* which imply a *phase transition* to unpredictable states. An understanding of nonlinear relationships is essential in the case of climate change, where our linear thinking does not do justice to the complexities and dynamics around CO_2_ emissions and warming. Positive meteorological, ecological, economic, social, psychological and behavioural feedback loops are leading to climate change with multiple potential tipping points that can radically change the conditions for life on Earth (Dodds 2011). History has seen collapses of civilization driven by environmental damage, resource depletion, climate change, hostile neighbouring cultures and inadequate societal responses, e.g. in the Mayan cities in Central America, Easter Island in the Pacific Ocean or Minoan Crete in Europe (Diamond 2006). Anthropogenic climate change and its impacts take place globally, in *nested, coevolving systems* at all scales from the planetary to the genetic and psychological.

This thinkbite follows CAS’s suggestion to reassemble culture, society and education in conversation with other systems between anarchy and stagnation—at the *edge of chaos* (Spannring and Hawke 2022). It is based on the authors’ encounter with young people’s experiences with the climate crisis on the *political level* and *personal level* (Spannring and Waechter 2023). As the next section discloses, these examples can be reinterpreted as a struggle with this same (un)dynamic position in nested systems. In the final section, the authors discuss the implications for the *institutional level* of the education system in light of the complexity turn in education science (e.g., Davis and Sumara 2006; Jacobson and Wilensky 2006; Hager and Beckett 2019; Kaplan and Garner 2020) and sustainability education (Sterling 2001). While drawing on research findings from environmental and climate change education^1^, we suggest—along with many environmental education researchers—that climate change not only raises questions about what and how to teach, but challenges education as a whole. The thinkbite thus aims to contribute conceptually and based on the complexity turn for more wholesome and creative responses to the climate challenge in the education system with all its subsystems.

## Young people’s experience with complexity

### Participation: dynamics and nested systems

Since the First World Climate Conference under the umbrella of the UN in 1979 in Geneva, Switzerland, and until the release of the 2019 Emissions Gap Report (UNEP 2020), international efforts to combat climate change and its associated effects have been largely ineffective, with little impact on national politics and policy-making. The IPCC 2022 is not much more hopeful. There has been some but only incremental adaptation in natural and social systems with gaps between the current adaptation and adaptation needs remaining across sectors and regions, especially under medium and high warming levels (IPCC 2022, p. 68). There is increasing evidence on limits to adaptation resulting from the interaction between adaptation constraints and the speed of change (ibid., p. 84) as well as evidence of increasing maladaptation. Inappropriate political responses to climate change “create long-term lock-in of vulnerability, exposure and risks that are difficult and costly to change and exacerbate existing inequalities for Indigenous Peoples and vulnerable groups, impeding achievement of SDGs, increasing adaptation needs and shrinking the solution space. Decreasing maladaptation requires attention to justice and a shift in enabling conditions towards those that enable timely adjustments for avoiding or minimising damage and for seizing opportunities” (ibid., p. 85). The IPCC assessment report calls for a “deep-rooted transformational adaptation” that creates new possibilities for adapting to the impacts and risks of the climate crisis by changing the central characteristics of the system, such as goals and values, and tackling the root causes of vulnerability. Five system transitions are necessary for a just and climate-resilient future. They pertain to the societal, energy, land and ocean ecosystems, urban and infrastructure, and industrial sectors and involve “shifts in most aspects of society” (ibid., p. 99).

With the Fridays for Future school strikes young people around the world have expressed their demands for quicker, more courageous and effective responses to the climate emergency. Their despair and anger over the inertia of political systems, and their search for social identity, political efficacy, and structural embeddedness were a driving force for young people’s politicisation (Shafi and Ran 2021; Spannring 2021; Spannring and Hawke 2022) that had some ripple effect on publics and political systems. A survey in Switzerland found that the public, by and large, perceived Greta Thunberg and the FFF movement positively and that Greta Thunberg (30%) and the FFF activism (23%), respectively, positively influenced their environmental concern and behaviour. Existing pro-environmental behaviour was reinforced and some of the unconvinced have to some extent also been reached (Fritz et al. 2023). In Germany and Austria, the dynamics captured the public debate around the European parliamentary election in 2019, benefitted the Green parties, and led several communities to declare a climate emergency. Here, we can see a case of self-organisation, emergence, and a return from stagnation to the edge of chaos where change becomes possible (Spannring and Hawke 2022). Against more traditional sociological understandings of agency, complex change is not affected by agents purposefully influencing the world in a mechanistic way, but by individuals’ behavioural modifications that can lead to large-scale transformation across nested systems (cf. Urry 2003, p. 106). However, the example of the Friday for Future school strikes also demonstrates how dynamics can be quickly reined in by other dynamics such as the Covid pandemic, the Russian war on Ukraine, and the massive inflation.

Studies on young climate activists’ self-perception illustrate further how the individual agent or group remains a cogwheel in a larger gearbox, i.e. part of a nested system, where some systems co-evolve more easily than others. Waechter and Steinmann (2024), for example, conclude that German and Austrian climate protesters “perceive themselves as influential role models of a climate-friendly lifestyle that is adopted by others. They also notice an (indirect) influence of the FFF movement on politics and governments as well as on the problem awareness of the population, but overall, their activism seems to be driven more by hope than by tangible results.” (2024, p. 85).

I can see that people are talking about the climate crisis everywhere and that this topic has really made it everywhere by now, that everyone is talking about it and that a rethink has simply taken place in many people’s minds or is currently taking place (…) and even if we haven’t achieved much at a political level yet, I realise that we have achieved a great deal at a social level. (FFF-activist, ibid., p. 10)

Politicians often say that FFF is great, invite us to talks and then twist and turn the issue so that something else comes out. For example, “Yes, we all need to drive a lot less”—which wasn’t actually the issue at all (…) and it’s simply shifted to the individual. (FFF-activist, ibid., p. 9)

Read from a CAS perspective, the first quote hints at the dynamics of collectives. As other animals with a strong predisposition to imitate others, human groups can be nudged in certain directions by a small number of partisans. Adaptive dynamics follow mutations such as novel behaviour or attitudes as “individuals come into contact with others on a specified social network, they alter their attitudes, their actions and even their normative groups, based on the attitudes, actions and normative groups of the others” (Levin 2006, p. 331). The second quote highlights that phenomena in complex, adaptive systems constitute wicked problems that cannot be solved like repairing a broken machine. Rather, they involve a meshwork of issues and constraints, different stakeholders with varying interests, values and problem definitions (Hawke and Pálsson 2017). There is no objective version of the problem, no objectively “right” answer, and no once and for all solution. Constraints and participants change and the problem-solving process does not end when the perfect solution is reached but when resources are exhausted (Madron and Jopling 2003). Differing perspectives on the issue of climate change are apparent in national party politics but also on the international level where drivers such as economic competition and the ever-increasing fight for resources override cooperation. No wonder robust national climate protection policies are stifled and the Kyoto Protocol failed to gain support from major players in a global common, in which individual agents—be they people, corporations or nations—act largely in their self-interest under different value-sets and constraints, and markets fail to account for environmental degradation.

### Learning at the edge of chaos

The first global school strike in March 2019 involved 1.6 million protesters worldwide and in September 2019, 7.6 million participants took part suggesting that the FFF movement is the largest globally coordinated climate protest in world history (Spannring and Hawke 2022; de Moor et al. 2021; Spannring 2021). However, this does not provide us with a complete picture of young people’s experiences with climate change. Participation in climate activism is shaped by cultural, structural and biographical affordances as well as by the perception of individual and collective self-efficacy (Prendergast et al. 2021), knowledge, attitudes and coping strategies (Ojala 2012). Furthermore, young people can express their climate concerns in various ways. Kuthe et al. (2019) identified four groups in a survey of 792 teenagers in German and Austrian schools. The Charitables, the biggest group with 40%, are the best informed with the lowest level of concern but a high level of climate-friendly behaviour in their everyday lives. 21% are Concerned Activists. They demonstrate the highest level of awareness of climate change and climate-friendly behaviour, and try to influence their friends and parents. However, they know little about climate change. The second biggest group (25%) are the Disengaged, who are not concerned about climate change, are not willing to act and do not know much about climate change. Finally, there are the Paralysed (14%), showing strong concern but neither engaging in climate-friendly behaviour nor multiplicative actions (ibid., p. 176 f.). Thus, almost 40% of the sample appear overwhelmed by the crisis and feel helpless. Globally, climate-related distress is substantial, with children and young people being acutely vulnerable. The Global Gen Z Study carried out in 31 countries, shows that climate change and environmental issues are among young people’s biggest concerns worldwide (Bogueva et al. 2024). Similarly, Hickman et al. (2021) report that in a comparative study of 16–25-year-olds in 10 countries 59% were very or extremely worried and more than 50% stated that they felt sad, anxious, angry, powerless, helpless, and guilty.

Ecopsychology provides insights into psychosocial drivers of climate change such as identities, everyday cultures and practices, consumer ideologies, an anthropocentric “sense of entitlement” (Vetlesen 2021), cognitive biases towards short-term gains at the expense of long-term goals, particularly at uncertain times, and the dilemma of the commons. The psychosocial impacts of climate change include anxieties, ontological insecurity, cognitive dissonance, and denialism (Norgaard 2011). The following quote from an interview with a 17-year-old student in upper secondary education reveals the ecological threat arising from the loss of a safe “holding environment” (Nicholsen 2003, quoted in Dodds 2011, p. 55) on the levels of nature, culture and personal life chances.

My cousin is 2 years old. They’ll never see it, they won’t have any more snow, like I experienced in my childhood. (…) I’m a ski instructor and work in the catering trade. You can see that the snow conditions are changing. You might not realize it, but it’s already clear that tourism can’t go on like this. (…) And the fear that my opportunities will be so limited in the future that it will no longer be possible to live the dreams I have. (non-activist female^2^, Spannring and Waechter 2023)

Traditional environmental campaigns aiming at frightening citizens into action are most likely counterproductive. High levels of threat without an adequate ability to cope leads to minimizing danger, denialism, scapegoating, anti-environmentalism, belief in conspiracy theories, displacement activities, pseudo-solutions and—on the other end of the spectrum—superego moralism (Dodds 2011, p. 42 ff.). Climate chaos and our propensity to cope with anxiety by clinging rigidly to the order of our old ways set off a positive feedback loop that further aggravates the crisis. In the language of CAS, the climate crisis represents a dramatically changing *fitness landscape*, to which social and psychological systems must adapt creatively by allowing for more non-orderly space rather than avoiding it (ibid., p. 172). Another 17-year-old student in upper secondary education expressed this edge of chaos quite aptly:

“Crisis” refers to situations in which something has to change, where there are major difficulties, whether in the personal or global, world political sphere. There are always many factors involved in a crisis that need to be changed. (…) It’s just a difficult situation where you have to learn. During the corona crisis, we didn’t immediately know what to do, what was right and what was wrong, and where we could help. (non-activist male, Spannring and Waechter 2023)

Such a probing, iterative search in a shared learning process is well captured by transformative learning theory, which stresses the crisis that occurs when old practices, beliefs and expectations are no longer valid and the consequent need for social and emotional support (Mezirow et al. 2000). Psychoanalyst Margy Sperry draws attention to “the phenomenology of complexity, that is, the feeling of living in and with the irreducible complexity of human experience, of being open to novelty, and of embracing the vulnerability that our human existential uncertainty entails” (2016, p. 349). Particularly in the case of the climate crisis, which confronts us with an existential threat and our limits of knowledge, we must bear to live and learn at the edge of chaos. It is where systems are poised for change by being open enough for change yet ordered enough to sustain the change (ibid., p. 356) and creativity is most potent.

Unfortunately, teachers are badly equipped with critical emotional awareness and ways to cope (Ojala 2023) to support learning at the edge of chaos. Education systems on all levels from policy and institutions to expected learning outcomes exert a ‘“gravitational pull” towards certainty and agreement: towards stasis’ which greatly impedes self-organisation around changes (Kleiman 2011, p. 62.7). Further, traditional understandings of learning represent it as something done by individuals, as a relatively stable and enduring product, independent of context, replicable and propositional (Hager and Beckett 2019), that is as something relatively static or mechanistic rather than collective, creative and innovative (for such innovative approaches see e.g. Paulsen et al. 2022). How can education respond to young people’s hopes and fears and facilitate shared learning processes for the planetary community in its human and non-human diversity? This question will be pursued in the next section, where insights into the complexity of education and conditions for emergence may inspire a reimagination of pedagogy to meet the challenges of climate change.

## Discussion: complex emergence in education?

In the context of the climate crisis, education is recognised as playing a significant role. Under the title “Changing minds, not the climate: the role of education,” UNESCO stresses its importance in increasing “the climate change mitigation and adaptation capacity of communities by enabling individuals to make informed decisions. […] [It] provides the skills people need to thrive in the new sustainable economy, working in areas such as renewable energy, smart agriculture, forest rehabilitation, the design of resource-efficient cities, and sound management of healthy ecosystems. Perhaps most important, education can bring about a fundamental shift in how we think, act, and discharge our responsibilities toward one another and the planet” (2017, no pages). The IPCC, too, regards education as “vital for […] supporting climate-resilient development” (IPCC 2022, p. 108).

However, evaluations of climate change education on regional, national, and international levels have come to unsatisfactory results. Educational institutions and professionals are insufficiently prepared, and professionals’ training and career development as well as funding and policy priorities lack orientation towards the climate crisis. There seems to exist a stubborn mix of denialism, displacement activities, intentional ambiguity, and a refusal to unlearn and reconsider the purposes and practices of education, which Alan Reid calls “pyropedagogies” (Reid 2019, p. 771).

Research in environmental, sustainability, and climate change education has moved away from the simple and individualistic information-deficit model in favour of a recognition of behavioural complexity within dynamic ecosystems of relationships (Marcinkowski and Reid 2019) and holds learning settings and the education system accountable for change. Critiquing the unsustainability of modernist education with its mechanistic, dualistic and anthropocentric worldview, Steven Sterling suggests a co-evolution of education *for* change, i.e. practices bringing about change in a person, community or society, and education *in* change, i.e. “policy changes made to educational rationale, theory and practice that affect and may facilitate (or hinder) education for change” (Sterling 2001, p. 34). He sees education systems driven by the demands of global competition and economic “structural adjustment” and locked into traditional social and educational paradigms. Students are still largely educated to “compete and consume” rather than to “care and conserve” (ibid., p. 21), while the situation demands a transformative approach that fosters responsibility, flexibility, resilience, innovation, and ecological and systemic thinking.

Environmental educators concede that the calculation of changing people and thereby changing society did not work out because the larger educational system and still larger social, cultural and economic systems offset efforts by the individual teacher. It is therefore essential “to make the educational institution as far as possible a microcosm of the emerging sustainable society […]” (Sterling 2001, p. 33). This implies a transformative, constructive and participatory approach with a strong sense of emergence to both “education *for* change” and “education *in* change”. Similarly, Brent Davis and Dennis Sumara argue that “transformations of learning systems cannot be understood in linear or mechanical terms” but rather as “prompting complex emergence” (2006, p. 130 f.). They cite the industrial restructuring in the 1980s from top-down, fragmented production to bottom-up, distributed structures in response to a more volatile economy as an example of a “flexibly adaptive—that is, a more intelligent—system” (ibid., p. 133). Climate change exposes us to tremendous volatility and demands education for a future that can only partly be imagined, and must therefore move from simplicity to complexity.

The conditions for such a move toward complex emergence are to be found on the edge between internal diversity and redundancy, randomness and coherence, within enabling constraints. Diversity among the units or agents of a system is the basis for a multitude of possible responses, i.e. novel ideas or actions, to changing conditions. It cannot be decreed from above and will not be appreciated if the group task is inconsequential. Diversity needs to be balanced with redundancy, i.e. duplications of aspects that are necessary for shared activities such as language and purpose. However, individual and collective possibilities are not at odds, since there should be freedom to pursue individual interests in the service of group possibilities, but the group endeavour should not be reducible to particular interests. Learning occurs not only on the level of individuals but at all levels of organization through the exchange of ideas between neighbouring individuals and collectives. While it is important to provide appropriate structures and space to facilitate these interactions, desisting from controlling the outcome is essential. Consequently, constraints should not be prescriptive but rather proscriptive, defining unacceptable action, to tap into the “unexplored space of possibility” (Davis and Sumara 2006, p. 148).

Putting these CAS insights into educational practice, one step forward could be the European Open Schooling framework. It aims to foster a bottom-up transformation for educational innovation through the involvement of a diversity of stakeholders such as teachers, students and their families, local communities, research communities, civil society organisations, business and industry in real-life projects that are meaningful for the students and their communities. Going beyond an isolated project the framework facilitates a fundamental reimagination of pedagogy, school structures and culture (Sotiriou and Cherouvis 2017).

In conclusion of this thinkbite, harking back to young people’s fears and hopes we aimed to show that a sustainable and ethical future requires attention to the preservation of safe operating spaces on all levels from the planetary ecosystem, and global political systems, down to educational institutions, classrooms and individual learners as sociopsychological systems in their own right. As the IPCC warns us of socioecological tipping points we also need to be wary of drivers that push young people over the boundaries of their respective safe operating spaces by locking them into an outdated and rigid educational system and deserting them as they face climate chaos and existential fears. Greta Thunberg’s “How Dare You”-Speech could also be read as addressing educational practitioners and researchers, calling them to action in the service of complex emergence in schools, classrooms and communities as dynamic yet nurturing environments. It seems that many young people are already in their starting blocks, while others need a helping hand.

## Footnotes

1 A presentation of the field of climate change education is beyond the scope of this thinkpiece, but see e.g. Environmental Education Research, Volume 25, 2019—Issue 6: Climate Change Education and Research for an overview.

2 The quotes from non-activist young people are from a participative film project of bachelor students in education science (University of Innsbruck, winter term 2022/23) with upper-secondary school students on climate change.
